# Preparation of Ultrafine Co- and Ni-Coated (Ti,W,Mo,Ta)(C,N) Powders and Their Influence on the Microstructure of Ti(C,N)-Based Cermets

**DOI:** 10.3390/ma17081807

**Published:** 2024-04-15

**Authors:** Zaiyang Zhao, Pengmin Jia, Yuhui Zhang, Lili Ma, Jingjing Sun, Yiping Xu, Yurong Wu

**Affiliations:** 1 Fujian Province Key Laboratory of Functional Materials and Applications, Xiamen University of Technology, Xiamen 361024, China; z279160894@163.com (Z.Z.);; 2 School of Materials Science and Engineering, Xiamen University of Technology, Xiamen 361024, China

**Keywords:** spray-drying-in-situ carbothermal reduction, ultrafine Co- and Ni-coated (Ti,W,Mo,Ta)(C,N) powders, Ti(C,N)-based cermet, microstructure

## Abstract

The use of metal-coated ceramic powders not only effectively enhances the wettability of the metal–ceramic interface but also promotes a more uniform microstructure in Ti(C,N)-based cermets, which is advantageous for improving their mechanical properties. In this study, ultrafine Co- and Ni-coated (Ti,W,Mo,Ta)(C,N) powders were synthesized via the spray-drying-in-situ carbothermal reduction method. Subsequently, Ti(C,N)-based cermets were effectively fabricated using the as-prepared ultrafine Co- and Ni-coated (Ti,W,Mo,Ta)(C,N) powders. The impact of reaction temperature, heating rate, and isothermal time on the phase and microstructure of prepared powders was analyzed using X-ray diffraction (XRD), scanning electron microscopy (SEM), and transmission electron microscopy (TEM). Additionally, the microstructure of the as-sintered cermets was experimentally investigated. The findings reveal that the complete reduction of Co and Ni metal salts, pre-coated on the surface of (Ti,W,Mo,Ta)(C,N) particles, can be achieved through rapid heating (10 °C/min) in a specific temperature range (600–1000 °C) with an isothermal time of 3 h at a lower reduction temperature (1000 °C). The synthesized powders have only two phases: the (Ti,W,Mo,Ta)(C,N) phase and Co/Ni phase, and no other heterogeneous phases were observed with an oxygen content of 0.261 wt.%. Notably, the conventional core–rim structure was not dominant in the cermets obtained from the prepared Co- and Ni-coated (Ti,W,Mo,Ta)(C,N) powders. Moreover, the heterogeneous segregation effect of the Co/Ni coating on the ultrafine powder particles resulted in a finer microstructure than the traditional cermets with the same composition. However, the grain size is mainly in the range of 0.5–0.8 μm. The weaker residual stresses at the core and rim interfaces and the finer particle distributions could theoretically enhance the toughness of Ti(C,N)-based cermets, simultaneously.

## 1. Introduction

Ti(C,N)-based cermets, owing to their excellent overall performance, are extensively utilized as cutting tools for semi-finishing, finishing, and high-temperature engine parts [1,2,3,4]. These cermets exhibit higher red hardness, higher wear resistance, and better thermal stability compared to conventional WC–Co cemented carbides, offering advantages in terms of resource reserves [5,6]. Therefore, Ti(C,N)-based cermets are considered an ideal alternative to WC–Co in the field of wear-resistant materials and tool materials [7,8]. However, the wide range of applications of cermets is limited by their low toughness. To improve their toughness and strength, the primary research on Ti(C,N)-based cermets has focused on grain refinement [9,10] and the addition of nano-strengthened phases [11,12]. For example, by adding 50 wt.% ultrafine Ti(C,N) powders, Lin et al. [9] prepared cermets with improved mechanical properties and wear resistance, and Zhang et al. [12] showed that Ti(C,N)-based cermets with 2 wt.% nano-Al_2_O_3_ increased tool life by about 50%. In addition, solid solution treatment can improve the composition discrepancy between the core and rim phases, so it is also an important way to improve its performance [13,14]. It has been demonstrated that the flexural strength of cermets prepared from (Ti,W,Mo,Nb,Ta)(C,N) solid solution powders can be increased by approximately 10% [15,16,17].

More importantly, obtaining a homogeneous microstructure is the key to improving the toughness of cermets and maintain high hardness. However, when utilized as a raw material for the preparation of cermets through conventional wet milling methods, the hard-phase particles tend to aggregate and grow during the process. This can result in an uneven distribution of the binder and the hard phases. Therefore, solid solution powders combined with wet milling methods significantly deteriorate the mechanical properties of (Ti,W,Mo,Ta)(C,N)-based cermets. To address these issues, conventional individual raw powders are being replaced by synthetic metal-coated ceramic powders [18,19,20]. The application of such powders can effectively improve the wettability of the metal–ceramic interface while allowing better control of the microstructure homogeneity of Ti(C,N)-based cermets [18,19,20]. According to recent reports [21,22,23,24], several methods have been developed for the synthesis of Co/Ni-coated mixed powders. For instance, Zhou et al. [21] synthesized Ni-Mo/Ti(C,N)-coated powders through non-homogeneous precipitation and thermal reduction using a Ni(NO_3_)_2_·6H_2_O solution and (NH_4_)_6_Mo_7_O_24_·4H_2_O solution. Dios et al. [22] proposed a bottom-up approach in which nickel precursors were chemically reduced on the surface of Ti(C,N) grains using Ni(NO_3_)_2_·6H_2_O and N_2_H_4_·H_2_O solutions. Using a chemical co-precipitation coating, Xu et al. [23] proposed that Ni-coated mixed powders were obtained by depositing reaction-generated NiC_2_O_4_ on the surface of the mixed powders using NiCl_2_ and (NH_4_)_2_C_2_O_4_ as precursors, followed by hydrogen reduction. Additionally, electroless plating and sol–gel methods have been utilized to produce metal-coated ceramic powders [24].

However, the current coating processes for preparing metal-coated powders are complex, which makes it difficult to meet the requirements of mass production. Furthermore, the reports on the preparation of coated powders using solid solution powders as the matrix are quite limited. In this study, a novel industrial-friendly method for the preparation of composite powders, namely the spray-drying-in-situ reduction method, has been developed. With this method, the uniform ultrafine Co- and Ni-coated (Ti,W,Mo,Ta)(C,N) powders have been successfully prepared. Furthermore, the influence of the synthesized composite powders as raw materials on the microstructure of Ti(C,N) cermets is preliminarily discussed, especially focusing on the distribution of the binder and hard phase, and the distribution of particle size.

As is well known, the pre-solid solution is beneficial in reducing interfacial stress and thereby enhancing the strength and toughness of cermets. Additionally, metal-coated ceramic is advantageous for achieving a uniform distribution of the ceramic phase in the binder phase, inhibiting the agglomeration and growth of the ceramic phase, thus refining grain size and enhancing the hardness of cermets. Therefore, it can be anticipated that the utilization of Co- and Ni-coated (Ti,W,Mo,Ta)(C,N) powders prepared in this study as raw materials can facilitate the production of cermets with outstanding comprehensive performance including high hardness, high strength, and high toughness at the same time. Furthermore, the spray-drying-in-situ reduction method developed in this study represents an industry-friendly powder preparation approach. Consequently, this research also provides essential raw-material support for the mass production of high-performance ultrafine Ti(C,N)-based cermets.

## 2. Materials and Methods

### 2.1. Preparation of Ultrafine Co- and Ni-Coated (Ti,W,Mo,Ta)(C,N) Powders

The self-prepared (Ti,W,Mo,Ta)(C,N) powders (200~500 nm), denoted as (Ti,Me)(C,N), have been utilized as the matrix. In order to avoid introducing unnecessary ions into the system, the soluble salts Ni(CH_3_COO)_2_·4H_2_O (Macklin, Shanghai, China, 99.0%) and (CH_3_COO)_2_Co·4H_2_O (McLean, 99.5%) have been employed as the precursors of Ni and Co. The PEG-4000 (Sinopharm Chemical Reagent Co., Shanghai, China, analytical purity) was used as both the surfactants and carbon sources.

**Figure 1:**
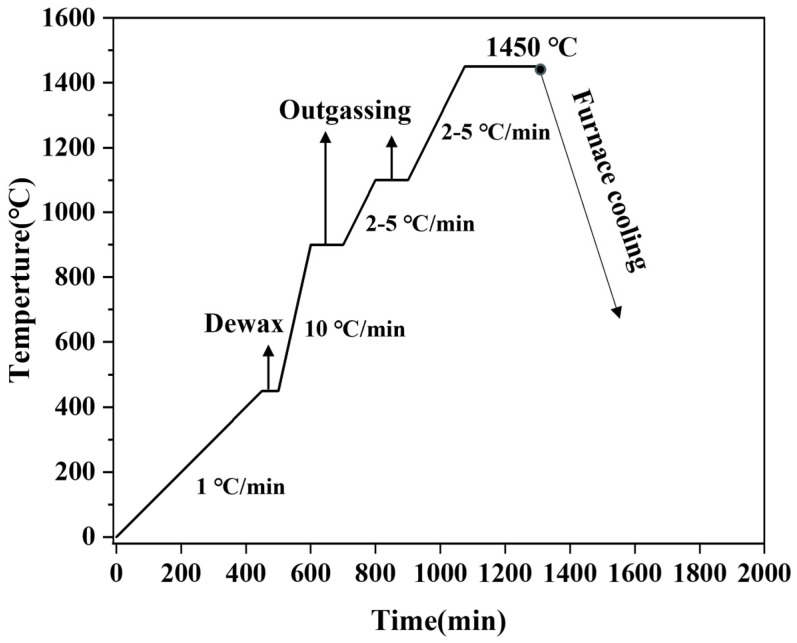
Sintering procedure for Ti(C,N)-based cermets under vacuum.

In this study, ultrafine Co- and Ni-coated (Ti,W,Mo,Ta)(C,N) powders were prepared by the spray-drying-in-situ carbothermal reduction method. Firstly, in order to eliminate agglomeration and obtain mono-dispersed (Ti,Me)(C,N) powders, the self-prepared (Ti,Me)(C,N) powders with 4 wt.% PEG-4000 as the surfactant were ground with carbide balls in a rolling ball mill for 12 h at a grinding speed of 190 rpm with a ball-to-material ratio of 10:1 in deionized water. Then, the weighed Ni(CH_3_COO)_2_·4H_2_O and (CH_3_COO)_2_Co·4H_2_O were added to the ground slurry. Herein, the Co and Ni content required for coating is 15 wt.%. To facilitate the complete dissolution and even distribution of the soluble salt, the slurry was continuously ground at 190 rpm for 1 h. Next, the slurry was sieved with a pore size of 0.062 mm. Eventually, the precursor powder was obtained via spray granulation at 110 °C at the outlet of the spray-drying tower. Herein, the introduction of spray drying is critical and the instantaneous drying property allows the soluble salt to be uniformly coated on the surface of the (Ti,Me)(C,N) particles. The reduction temperature, heating rate, and isothermal time of the in-situ carbothermic reduction process of the precursor were then systematically investigated. The prepared powders were crushed and sieved using a Mesh-80 sieve. Then, the sieved powders were subsequently compacted into sample strips under 250 MPa. Finally, Ti(C,N)-based cermets were sintered at 1450 °C for 1 h under 10^−1^ Pa in the furnace. The sintering curve is displayed in Figure 1. The nominal composition of the cermets is as follows: (Ti,Me)(C,N)-7.5 wt.%Co-7.5 wt.%Ni. To compare, traditional Ti(C,N)-based cermets were prepared using the same nominal composition by individual raw powders (Ti(C,N), WC, Mo_2_C, TaC, Co, and Ni), and sintering parameters. The average grain size was determined using the lineal intercept method by counting approximately one thousand hard-phase particles based on SEM images.

### 2.2. Characterization

Phase identifications were conducted via XRD (Smartlab 3 kW, Rigaku, Tokyo, Japan) using Cu Kα radiation at a step rate of 0.02°/s, over a 2θ range of 30° to 90° with a scanning speed of 1° per minute. The numerical analysis of XRD patterns was conducted using MDI JADE (Edition 6.5, Materials Data Ltd., Livermore, CA, USA). The oxygen content of samples was measured using the Oxygen/Nitrogen/Hydrogen Analyzer (LECO, ONH836, St. Joseph, MO, USA), calibrated against a standard with an oxygen content of 0.0041%. The morphology of experimental powders at various stages and the sintered cermet specimens was characterized through SEM (Sigma 500, ZEISS, Jena, Germany) coupled with EDXA. For SEM observations, the Inlens mode was employed to examine the morphology of powders, while the Backscattered Electron (BSE) mode was utilized to observe the microstructure of the cermets, with magnifications of 2000×, 5000×, 10,000×, and 20,000×. TEM and HRTEM analyses were performed on a Talos F200S instrument to test the element mapping, lattice parameters, and interface structure of the solid solution powders and the coated powders at the 200 kV (FEI) accelerating voltage.

## 3. Results and Discussion

### 3.1. Ultrafine (Ti,W,Mo,Ta)(C,N) Powders

**Figure 2:**
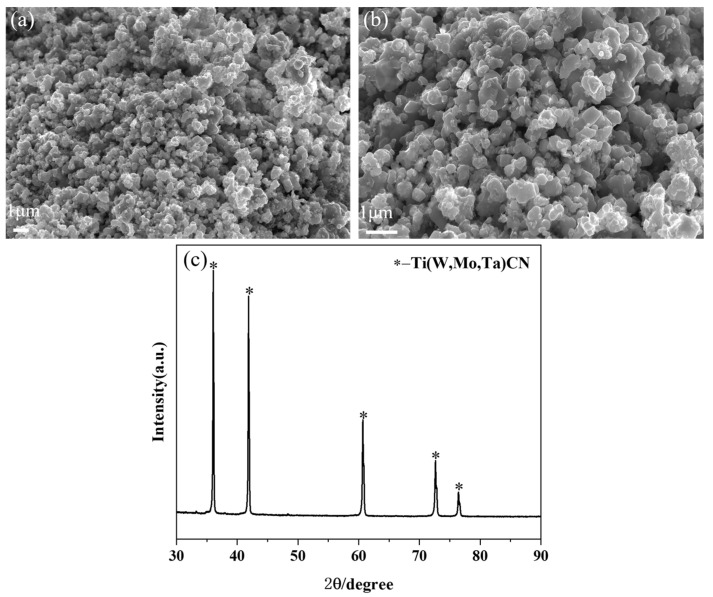
Morphology and XRD patterns of the (Ti,Me)(C,N) powders: (**a**,**b**) SEM images; (**c**) XRD patterns.

In this study, (Ti,Me)(C,N) powders were synthesized through a carbothermal reduction–nitridation reaction. As is depicted in Figure 2a,b, the prepared (Ti,Me)(C,N) powders have a relatively uniform size of around 200 nm. Figure 2c displays the XRD pattern of the prepared (Ti,Me)(C,N) powder. Notably, only a single phase was observed.

**Figure 3:**
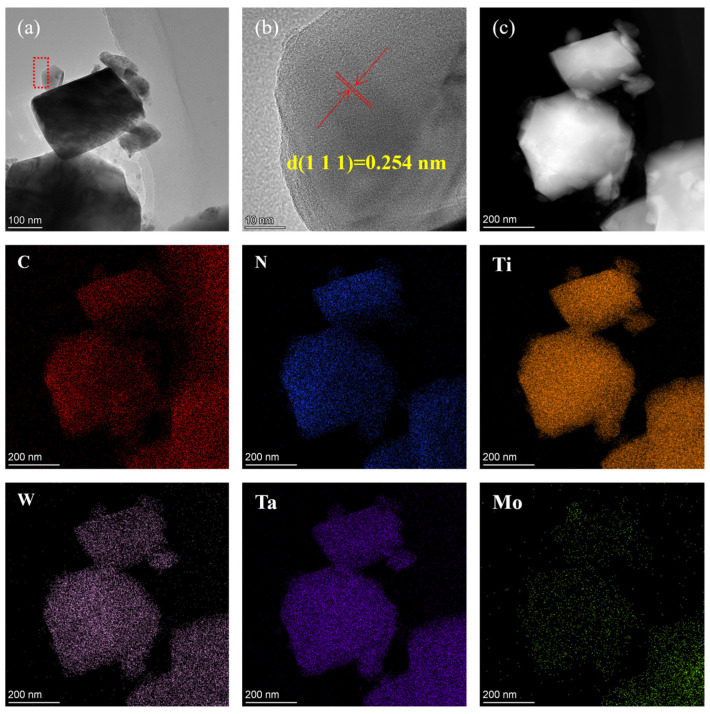
TEM analysis of the prepared (Ti,Me)(C,N) powders: (**a**) TEM image; (**b**) HRTEM image of the red dotted section in panel (**a**); (**c**) HAADF image; and the corresponding EDS compositional mappings.

**Figure 4:**
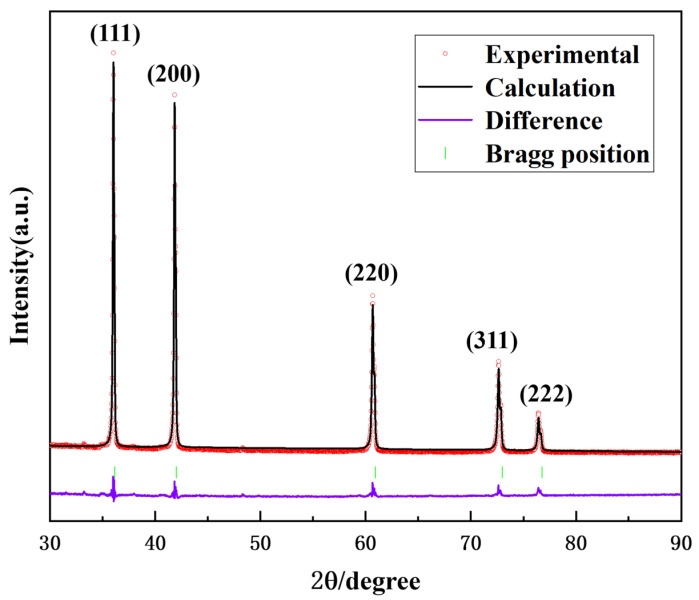
Rietveld refinement of (Ti,Me)(C,N) powders.

To further determine the phase crystal structure of the prepared (Ti,Me)(C,N) powders, high-resolution transmission electron microscopy (HRTEM) was applied, as depicted in Figure 3a,b. The solid solution powder exhibits a typical periodic atomic arrangement, with (111) interplane distances of 0.254 nm. Figure 3c presents the high-angle annular dark-field (HAADF) images and the corresponding energy-dispersive X-ray spectroscopy (EDS) composition maps of (Ti,Me)(C,N) powders. As is presented in the results, the distribution of each metal element is uniform, and no segregation is observed. Figure 4 displays the Rietveld refinement analysis of the XRD results, which identifies the space group as Fm3m, and the (111) interplane distance as 0.256 nm. The lattice parameter determined through Rietveld refinement shows a 0.7% discrepancy compared with the HRTEM result, verifying the obtained powder as a single-phase (Ti,W,Mo,Ta)(C,N) solid solution carbonitride.

### 3.2. Precursor Powder

**Figure 5:**
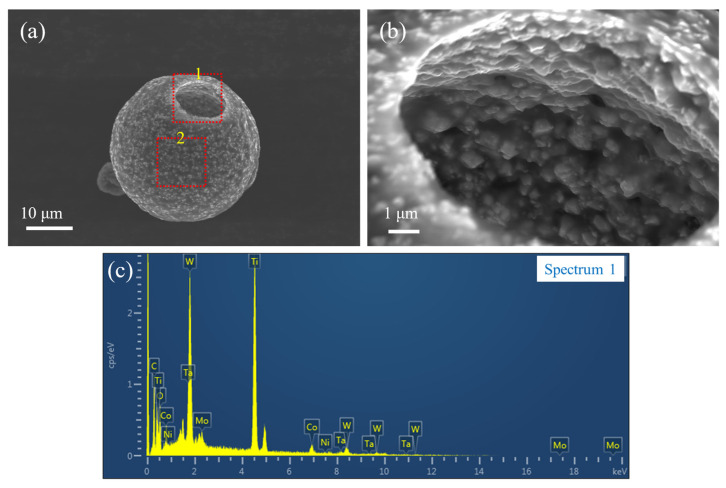
Morphology and EDS spectrum of the precursor powders: (**a**) Precursor powder; (**b**) Magnified image of the area shown in box 1 in the panel (**a**); and (**c**) EDS spectrum of the surface in box 2 in the panel (**a**).

Compared to the original solid solution powder (see Figure 2a,b), the SEM images of the spray-dried precursor powder in Figure 5a,b reveal a significant morphological transformation after spray drying. The precursor powders display a hollow spherical structure. This can be attributed to the rapid drying rate during spray drying [25]. In addition, the solid solution powder particles in Figure 5a,b are observed to be enveloped and bonded together by the transparent metal salt. Based on the SEM and EDS analyses, the metal salts were uniformly distributed on the surface of the (Ti,Me)(C,N) powders after spray drying. This outcome not only demonstrates the efficacy of the spray-drying technique in modifying powder morphology but also highlights its potential in facilitating the uniform coating of metal salts on powder surfaces. Such structural and compositional modifications are pivotal for enhancing the functional properties of the powders for their subsequent applications in various technological fields.

### 3.3. Phase Evolution and Microstructure of Coated Powder

#### 3.3.1. Influence of Reaction Temperature

Based on the thermodynamic analysis by Liu et al. [26], the reduction temperatures for in situ carbothermal reduction were set between approximately 900 and 1300 °C. In this study, 900 °C, 1100 °C, and 1300 °C were selected for research, with a heating rate of 2 °C/min and an isothermal time of 1 h.

**Figure 6:**
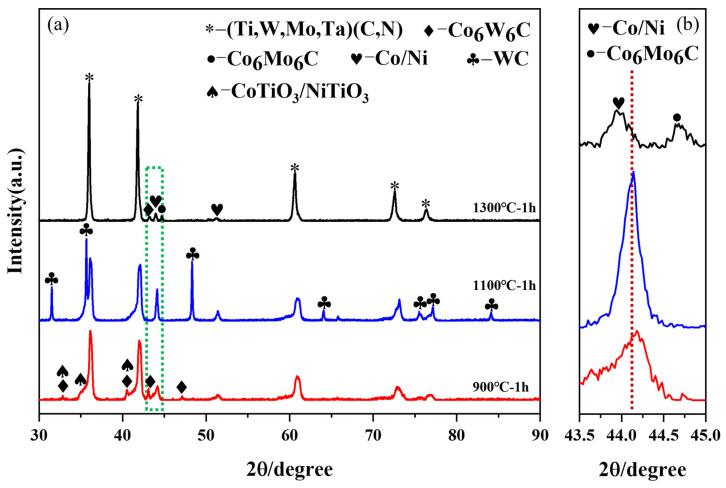
XRD patterns of precursor powders after reduction at different temperatures: (**a**) full spectra; (**b**) partial enlarged spectra.

Firstly, all the reduced powder samples were analyzed by XRD. Figure 6a shows the XRD patterns of the powders obtained at three reduction temperatures and Figure 6b displays an enlarged view of the characteristic peak (111) of Co/Ni. From Figure 6a, it can be seen that all three samples have the Co/Ni phase except the (Ti,Me)(C,N) solid solution phase. This means that the reduction reactions of cobalt salt and nickel salt have already begun from 900 °C [26]. However, the appearance of the CoTiO_3_ and NiTiO_3_ phases indicates that the reaction temperature of 900 °C is too low, and the oxidation–reduction reaction is not complete. In addition, there was still another phase, Co_6_W_6_C, in the sample at 900 °C and the WC phase for the sample of 1100 °C. The presence of CoTiO_3_, NiTiO_3_, Co_6_W_6_C, and WC phases also indicates the occurrence of an oxidation–decomposition reaction during the in-situ reduction process of (Ti,Me)(C,N). Upon further elevation of the reaction temperature to 1300 °C, two decarburized phases, namely Co_6_W_6_C and Co_6_Mo_6_C, were observed in the powder. Furthermore, in comparison to the samples at 900 °C and 1100 °C, a noticeable shift in the peak position of the Co/Ni phase was observed for the samples treated at 1300 °C (Figure 6b). This shift indicates the occurrence of the solid-phase sintering phenomenon at this temperature, leading to substantial incorporation of W and Mo into the Co/Ni phase [10]. Consequently, it can be inferred that a reduction temperature of 1300 °C is excessively high.

**Figure 7:**
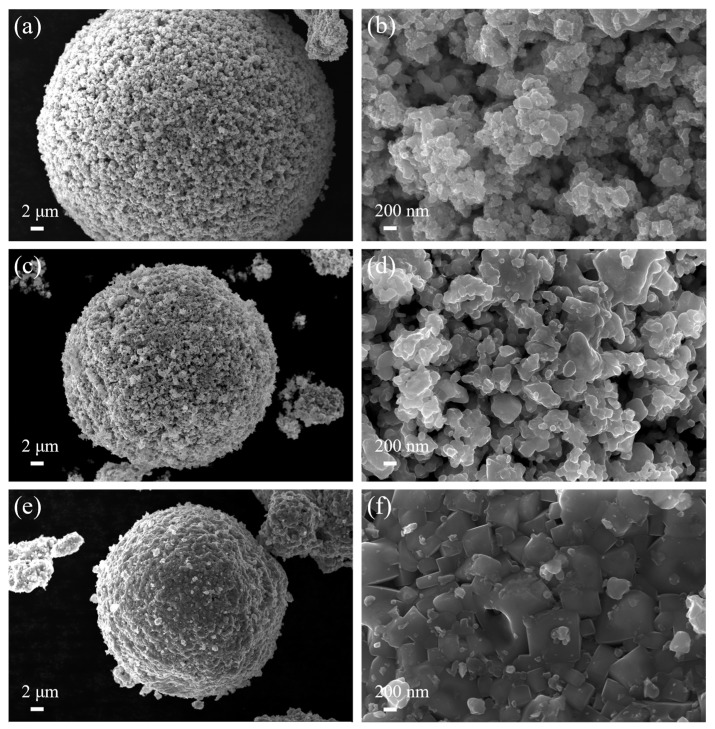
Morphology of coated powder obtained by reduction at different temperatures: (**a**,**b**) 900 °C, 1 h; (**c**,**d**) 1100 °C, 1 h; and (**e**,**f**) 1300 °C, 1 h.

Figure 7 displays the SEM morphology of the reduced samples. After in-situ reduction at 900 °C, the solid solution particles were coated by numerous particles with a size of approximately tens of nanometers (Figure 7a,b). According to the XRD results, it becomes evident that these nanoparticles adhering to the surface of solid solution particles correspond to Co/Ni. Notably, the number of small particles attached to the solid solution particles becomes less when the reduction temperature reaches 1100 °C (see Figure 7c,d), and there is a slight sintering phenomenon. As the reduction temperature rises to 1300 °C (Figure 7e,f), sintering is evident on the powder surface, resulting in the sintering and fusing of particles. This phenomenon is attributed to the high reduction temperature. The Co/Ni phase undergoes reduction first, subsequently reaching the melting point and starting to liquefy, promoting sintering. This is confirmed by XRD analysis.

**Figure 8:**
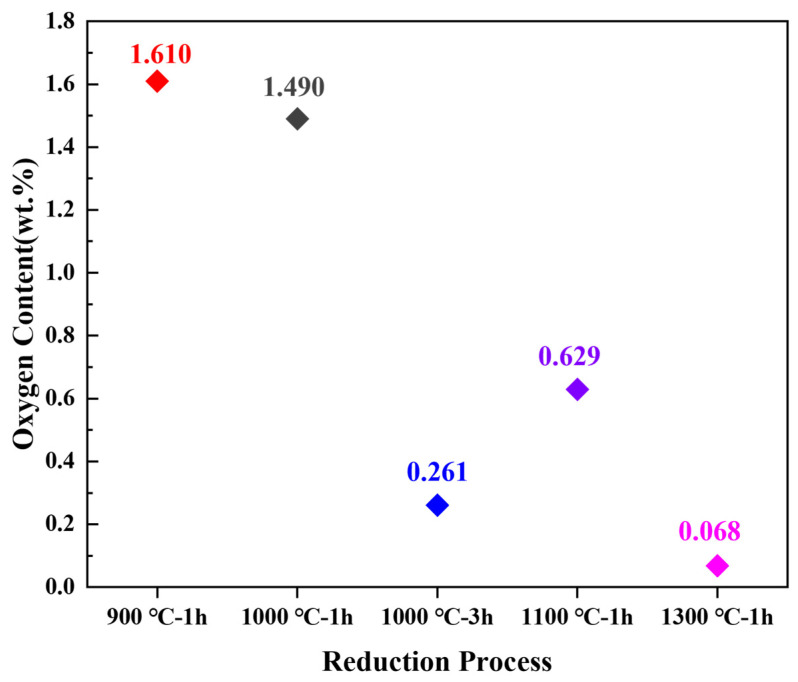
The oxygen content of the reduced powders under different conditions.

Notably, oxygen content is one of the most intuitive indicators of the completeness of reduction [27]. As is delineated in Figure 8, the oxygen content of the three samples, subsequent to reduction at varying temperatures, exhibits a discernible downward trend with an increase in temperature. The sample reduced at 1300 °C exhibits the lowest oxygen content (0.068 wt.%); however, XRD and SEM results indicate significant sintering of the powder at this temperature. The sample obtained at 900 °C demonstrates a favorable coating state, yet XRD and oxygen content analyses reveal an inadequate reduction temperature and incomplete reduction reaction. The 1100 °C sample possesses a relatively low oxygen content, but the presence of the WC phase and slight sintering phenomenon suggest that further optimization research is required for the reduction process.

#### 3.3.2. Influence of Isothermal Time

The formation of intermediate phases such as WC and Co_6_W_6_C can significantly affect the properties of the coated powder, making it less suitable for the subsequent steps involved in cermet preparation and sintering [28]. To mitigate the formation of these intermediate phases, a strategy of rapid heating at a rate of 10 °C/min within the temperature range of 600 °C to 1000 °C was employed. This approach aimed to reduce the reaction time, thereby minimizing the chance for intermediate phases to develop. Furthermore, based on the aforementioned findings regarding the reduction reactions at various temperatures, a relatively low reduction temperature of 1000 °C was further investigated. Particularly, the influence of the isothermal time at the reduction temperature on the reduction effect has been systematically investigated in this study.

**Figure 9:**
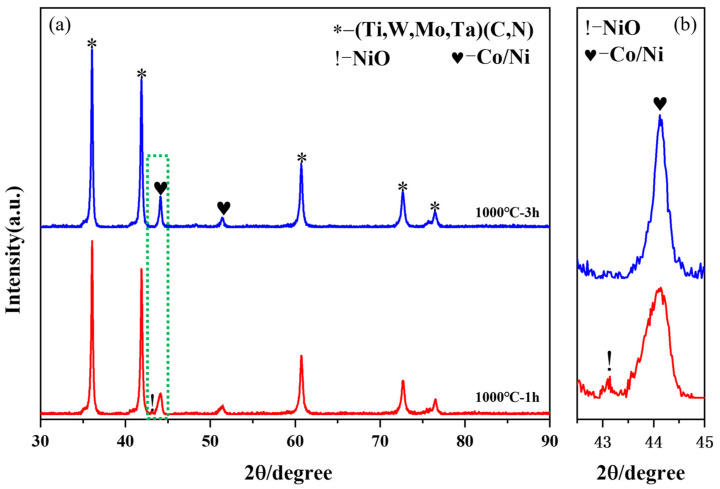
XRD patterns of precursor powders after reduction at different isothermal times: (**a**) full spectra; (**b**) partial enlarged spectra.

Figure 9a depicts the XRD patterns for the samples reduced at 1000 °C with different isothermal times, 1 h and 3 h (named 1000 °C, 1 h and 1000 °C, 3 h). The absence of WC and Co_6_W_6_C phases in Figure 9a compared to Figure 6 indicates that the rapid heating effectively inhibits the occurrence of a solid solution oxidation–decomposition reaction. The presence of the weak metal oxide peak in the 1000 °C, 1 h sample (Figure 9b) suggests that the reduction reaction of the metal salt remains incomplete. However, when the isothermal time was extended to 3 h, not only did the metal oxidation phase disappear, but the relative strength of the Co/Ni phase also increased (as depicted in Figure 9b). This observation suggests that apart from reduction temperature, isothermal time also exerts an influence on the reduction reaction of the metal salt. In practical applications, a relatively low reduction temperature with an extended isothermal time can be employed to achieve powder products with uniform dispersion instead of sintering agglomeration.

**Figure 10:**
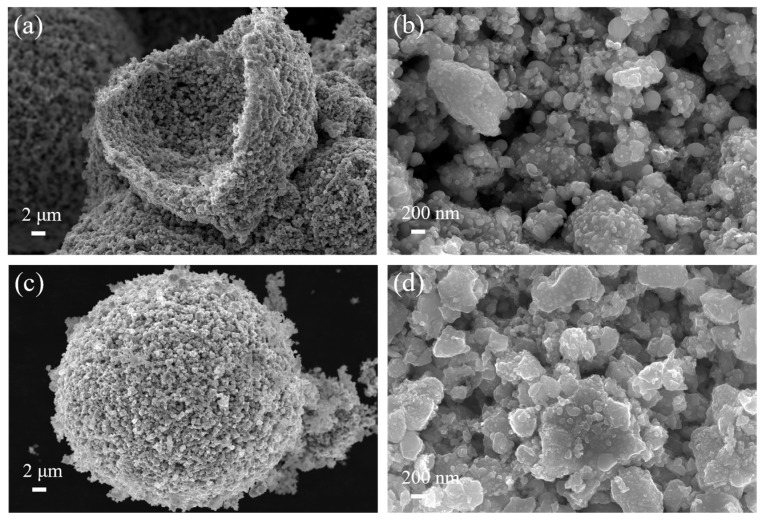
Morphology of coated powder obtained by reduction at different isothermal times: (**a**,**b**) 1000 °C, 1 h; (**c**,**d**) 1000 °C, 3 h.

Figure 10 displays the SEM morphology of sample 1000 °C, 1 h and 1000 °C, 3 h. From Figure 10a,c, the reduced samples still display a morphology similar to that of the spray-dried precursors—hollow spheres at low magnification. The magnified images (Figure 10b,d) show that the carbothermal reduction occurs mainly on the surface of the metal-salt-coated mixed powder particles. Numerous nanometer-sized (~50 nm) Co/Ni particles were attached to the surface of solid solution particles.

The oxygen content of the 1000 °C, 1 h and 1000 °C, 3 h samples is also displayed in Figure 8. The results reveal that the coated powders reduced at 1000 °C for 3 h had the lowest oxygen content of 0.261 wt.%. This result also confirms the XRD analysis result indicating a more complete reduction reaction. In contrast, the oxygen content of the coated powders maintained at 1000 °C for 1 h was 1.490 wt.%. This also confirms the results of XRD.

**Figure 11:**
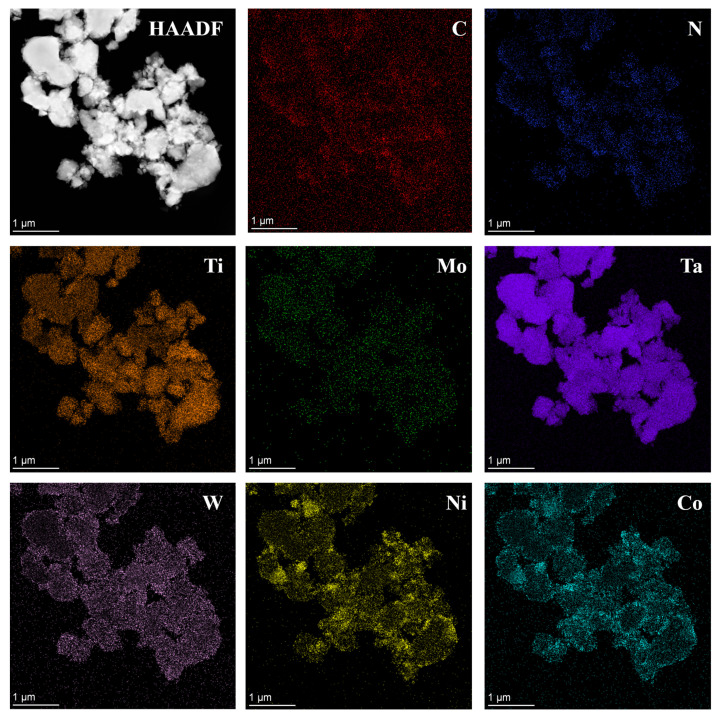
TEM analysis of the coated powders after isothermal time at 1000 °C for 3 h: HAADF image and the corresponding EDS compositional mappings.

As is depicted in Figure 11, HRTEM tests were performed to further verify the structure of the coated powders held at 1000 °C for 3 h. The element mappings display a highly homogeneous distribution of Co and Ni elements on the surface of the solid solution particles, with no segregation or aggregation. Moreover, the Co and Ni elemental spectrums have slightly larger profiles than the solid solution particles, further demonstrating that Co and Ni are uniformly coated on the surface of (Ti,Me)(C,N) particles, showing a typical core-shell structure.

**Figure 12:**
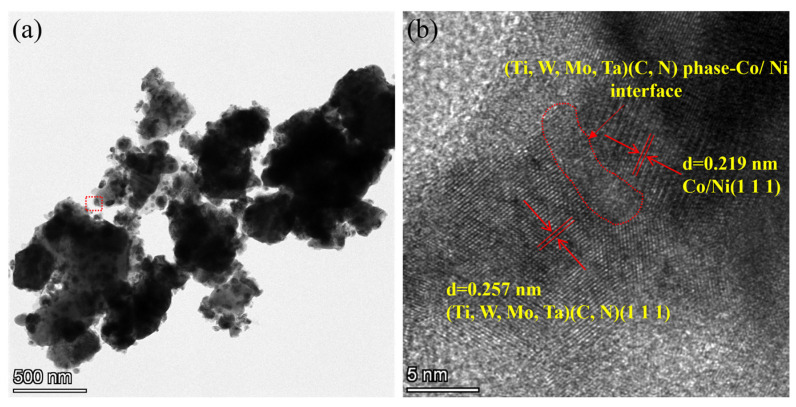
TEM analysis of the coated powders after isothermal time at 1000 °C for 3 h: (**a**) TEM image; (**b**) HRTEM image.

Additional information regarding the (Ti,Me)(C,N) phase and Co/Ni phase can be obtained from the HRTEM analyses, as shown in Figure 12. Figure 12b depicts the state at the interface between the (Ti,Me)(C,N) phase and the Co/Ni phase. The results demonstrated that following reduction, Co/Ni are generated in situ on the surface of (Ti,Me)(C,N) particles. It is noteworthy that there is a significant interaction between the Co/Ni lattice and the (Ti,Me)(C,N) lattice. Thus, in conjunction with the findings of element mappings, it is evident that ultrafine Co- and Ni-coated (Ti,W,Mo,Ta)(C,N) powders were successfully prepared by subjecting the powders to isothermal treatment at 1000 °C for 3 h.

### 3.4. Microstructure of (Ti,W,Mo,Ta)(C,N)-Based Cermets

**Figure 13:**
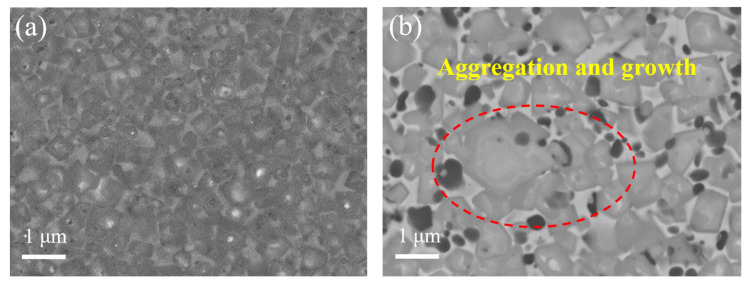
Microstructure of Ti(C,N)-based cermets: (**a**) cermets fabricated using the coated powder; (**b**) traditional cermets.

SEM analysis shows strong microstructural differences between the cermets fabricated using the coated powders and the traditional cermets (Figure 13). Traditional cermets have a typical core–rim structure and are embedded in a tough metallic binder phase, as shown in Figure 13b. Contrastingly, as is shown in Figure 13a, no obvious core–rim structure was found in the cermets fabricated using the coated powders. Furthermore, when conventional individual raw powders are employed for the preparation of cermets, the hard-phase particles tend to aggregate and grow up during the sintering process, as circled in red in Figure 13b. However, a relatively homogeneous distribution of hard-phase particles is observed for cermets fabricated using coated powder.

**Figure 14:**
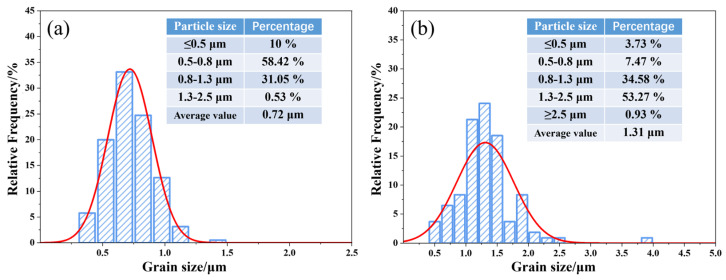
Particle size distribution and average grain sizes of Ti(C,N)-based cermets(The normal distribution curve for particle size is shown in red): (**a**) cermets fabricated using coated powder; (**b**) traditional cermets.

The particle size analysis of the prepared two cermets (Figure 14) reveals that traditional cermets, which are prone to hard-phase particle aggregation, exhibit an average particle size of 1.31 μm. In contrast, cermets fabricated using coated powders demonstrate a significantly finer average particle size of 0.72 μm, with a narrow distribution range (0.5–0.8 μm).

The underlying cause of these observed structural variations lies in the Ni and Co coatings. The homogeneous distribution of Ni and Co coatings produces a heterogeneous segregation effect on ultrafine powder particles, effectively reducing the tendency for hard-phase particle agglomeration and growth. In addition, the rim formation of the cermets fabricated using the coated powders is more effective than that of traditional cermets [23]. The rapid formation of rim phases further prevented the grain growth of hard particles [29]. Furthermore, the absence of a core–rim structure in cermets fabricated using the coated powders can be explained: due to the small particle size of the raw (Ti,W,Mo,Ta)(C,N) powder and the complete solid solution that has been achieved, this means that there are no remnants of the raw Ti(C,N) to form the core structure. Consequently, the entire hard phase constituted a homogeneous solid solution of (Ti,Me)(C,N), as indicated by reference [21]. Since the compositions of the core and rim phases of cermets fabricated using coated powders do not differ significantly, the residual stresses at the interface between the core and rim may be weakened [30]. Combined with the microstructure of fine and uniformly distributed particles, it is theoretically advantageous for enhancing the toughness of Ti(C,N)-based cermets [31].

In this chapter, the preparation process and conditions of Co- and Ni-coated (Ti,W,Mo,Ta)(C,N) powders have been systematically discussed. It is evident that the precursor powder with metal salts uniformly coated on the surface of the (Ti,Me)(C,N) particles can be obtained through the spray-drying process. Subsequently, in order to achieve complete in-situ reduction of the metal salts coated on the surface of the (Ti,Me)(C,N) particles, the effects of reduction temperature, heating rate, and isothermal time were investigated. The results suggest that the optimal reduction process is as follows: a reduction temperature set at 1000 °C and a rapid heating process (10 °C/min) adopted between 600 °C and 1000 °C with an isothermal time of 3 h. The coated powder obtained under these conditions exhibits a core-shell structure with Co/Ni nanoparticles uniformly coated on the surface of (Ti,Me)(C,N).

In order to validate the application effect of the prepared coated powders, this study also investigates the influence of the coated powders on the microstructure of Ti(C,N)-based cermets. The results indicate that using coated powder as raw materials can achieve a homogeneous distribution of the hard and binder phases in the microstructure with a finer grain size (~0.72 μm).

It should be noted that the preparation parameters mentioned in this article are based on laboratory data. In practical applications, it may be necessary to make appropriate adjustments due to variations in equipment and environmental conditions.

## 4. Conclusions

In summary, this paper presents a novel spray-drying-in-situ carbothermal reduction method to synthesis Co- and Ni-coated (Ti,W,Mo,Ta)(C,N) powders. Furthermore, the potential application of the synthesized powder in cermets is also discussed.

(1). Ultrafine Co- and Ni-coated (Ti,W,Mo,Ta)(C,N) powders were successfully prepared via the spray-drying-in-situ carbothermal reduction method. Herein, the precursors of Co/Ni were (CH_3_COO)_2_Co·4H_2_O, and Ni(CH_3_COO)_2_·4H_2_O. Firstly, the (Ti,Me)(C,N) particles were physically coated by the precursors of Co/Ni through spray drying. Then, the acetates were completely reduced in situ into Co and Ni. TEM and other analytical methods proved that the prepared powders displayed a core ((Ti,W,Mo,Ta)(C,N))-shell (Co/Ni) structure.
(2). Notably, the reduction reaction is facilitated by increasing the reduction temperature or extending the isothermal time. Moreover, the generation of intermediate phases can be avoided by rapid heating (10 °C/min) within a specific temperature range (600–1000 °C). It is feasible to achieve the complete reduction of Co and Ni metal salts pre-coated on the surface of (Ti,Me)(C,N) particles at a lower reduction temperature of 1000 °C with a long isothermal time of 3 h.
(3). The cermets fabricated using the prepared ultrafine Co- and Ni-coated (Ti,W,Mo,Ta)(C,N) powders demonstrated a more uniform and finer microstructure compared with traditional cermets. They lacked the conventional core–rim structure. The weaker residual stresses at the core and rim interfaces and the finer particle size could theoretically enhance the toughness and hardness of Ti(C,N)-based cermets, simultaneously.
(4). However, this article did not investigate the impact of ultrafine coated powders on the mechanical properties of Ti(C,N)-based cermets. According to the literature reports [10], the particle size of the raw materials directly influences the preparation process of cermets. Therefore, it is necessary to systematically study the preparation process of alloys in order to obtain optimal mechanical properties. The corresponding research will be systematically elaborated on in our subsequent studies.

Spray drying is widely utilized in the large-scale production of powders due to its exceptional drying efficiency and the high quality of the resulting powders. In this study, we propose for the first time a spray-drying-in-situ carbothermal reduction method for the preparation of metal-coated (Ti,W,Mo,Ta)(C,N) powders. This method is a simple process, with readily available raw materials and easy-to-use equipment. Based on the optimized reduction conditions obtained in this study, it is extremely feasible to achieve industrial-scale conversion of Co- and Ni-coated (Ti,W,Mo,Ta)(C,N) powders in practical applications. This provides essential raw material conditions for the development and large-scale production of high-performance ultrafine crystalline Ti(C,N)-based cermets.

## Data Availability

All data generated and analyzed during this study are included in this article.
